# Supplementary material for: A Critical View of the Application of the APEX Software (Aqueous Photochemistry of Environmentally-Occurring Xenobiotics) to Predict Photoreaction Kinetics in Surface Freshwaters
Source: Molecules. 2019 Dec 18;25(1):9. doi: 10.3390/molecules25010009 (PMC7017383; doi:10.3390/molecules25010009)
Supplement: Supplementary file 1 [file molecules-25-00009-s001.zip › 3.2.4_gcc-4.4.0.zip!/3.2.4_gcc-4.4.0/3.2.4_gcc-4.4.0/doc/octave/html/interpreter/String-Conversions.html]

String Conversions - Untitled


Next: Character Class Functions,
Previous: Manipulating Strings,
Up: Strings


---

### 5.6 String Conversions

Octave supports various kinds of conversions between strings and
numbers. As an example, it is possible to convert a string containing
a hexadecimal number to a floating point number.

```
     hex2dec ("FF")
          => ans = 255
```

— Function File: **bin2dec** (s)  
> Return the decimal number corresponding to the binary number stored
> in the string s. For example,
>
> ```
>           bin2dec ("1110")
>                => 14
> ```
>
> If s is a string matrix, returns a column vector of converted
> numbers, one per row of s. Invalid rows evaluate to NaN.
>
>
>
> **See also:** dec2hex, base2dec, dec2base, hex2dec, dec2bin.

— Function File: **dec2bin** (n, len)  
> Return a binary number corresponding to the non-negative decimal number
> n, as a string of ones and zeros. For example,
>
> ```
>           dec2bin (14)
>                => "1110"
> ```
>
> If n is a vector, returns a string matrix, one row per value,
> padded with leading zeros to the width of the largest value.
>
> The optional second argument, len, specifies the minimum
> number of digits in the result.
>
>
>
> **See also:** bin2dec, dec2base, base2dec, hex2dec, dec2hex.

— Function File: **dec2hex** (n, len)  
> Return the hexadecimal string corresponding to the non-negative
> integer n. For example,
>
> ```
>           dec2hex (2748)
>                => "ABC"
> ```
>
> If n is a vector, returns a string matrix, one row per value,
> padded with leading zeros to the width of the largest value.
>
> The optional second argument, len, specifies the minimum
> number of digits in the result.
>
>
>
> **See also:** hex2dec, dec2base, base2dec, bin2dec, dec2bin.

— Function File: **hex2dec** (s)  
> Return the integer corresponding to the hexadecimal number stored
> in the string s. For example,
>
> ```
>           hex2dec ("12B")
>                => 299
>           hex2dec ("12b")
>                => 299
> ```
>
> If s is a string matrix, returns a column vector of converted
> numbers, one per row of s. Invalid rows evaluate to NaN.
>
>
>
> **See also:** dec2hex, base2dec, dec2base, bin2dec, dec2bin.

— Function File: **dec2base** (n, b, len)  
> Return a string of symbols in base b corresponding to
> the non-negative integer n.
>
> ```
>           dec2base (123, 3)
>                => "11120"
> ```
>
> If n is a vector, return a string matrix with one row per value,
> padded with leading zeros to the width of the largest value.
>
> If b is a string then the characters of b are used as
> the symbols for the digits of n. Space (' ') may not be used
> as a symbol.
>
> ```
>           dec2base (123, "aei")
>                => "eeeia"
> ```
>
> The optional third argument, len, specifies the minimum
> number of digits in the result.
>
>
>
> **See also:** base2dec, dec2bin, bin2dec, hex2dec, dec2hex.

— Function File: **base2dec** (s, b)  
> Convert s from a string of digits of base b into an
> integer.
>
> ```
>           base2dec ("11120", 3)
>                => 123
> ```
>
> If s is a matrix, returns a column vector with one value per
> row of s. If a row contains invalid symbols then the
> corresponding value will be NaN. Rows are right-justified before
> converting so that trailing spaces are ignored.
>
> If b is a string, the characters of b are used as the
> symbols for the digits of s. Space (' ') may not be used as a
> symbol.
>
> ```
>           base2dec ("yyyzx", "xyz")
>                => 123
> ```
>
>
>
>
> **See also:** dec2base, dec2bin, bin2dec, hex2dec, dec2hex.

— Loadable Function: s = **num2hex** (n)  
> Typecast a double precision number or vector to a 16 character hexadecimal
> string of the IEEE 754 representation of the number. For example
>
> ```
>           num2hex ([-1, 1, e, Inf, NaN, NA]);
>           => "bff0000000000000
>               3ff0000000000000
>               4005bf0a8b145769
>               7ff0000000000000
>               fff8000000000000
>               7ff00000000007a2"
> ```
>
>
>
>
> **See also:** hex2num, hex2dec, dec2hex.

— Loadable Function: n = **hex2num** (s)  
> Typecast the 16 character hexadecimal character matrix to an IEEE 754
> double precision number. If fewer than 16 characters are given the
> strings are right padded with '0' characters.
>
> Given a string matrix, `hex2num` treats each row as a separate
> number.
>
> ```
>           hex2num (["4005bf0a8b145769";"4024000000000000"])
>           => [2.7183; 10.000]
> ```
>
>
>
>
> **See also:** num2hex, hex2dec, dec2hex.

— Function File: [num, status, strarray] = **str2double** (str, cdelim, rdelim, ddelim)  
> Convert strings into numeric values.
>
> `str2double` can replace `str2num`, but avoids the use of
> `eval` on unknown data.
>
> str can be the form [+-]d[.]dd[[eE][+-]ddd] in which
> d can be any of digit from 0 to 9, and [] indicate
> optional elements.
>
> num is the corresponding numeric value. If the conversion
> fails, status is -1 and num is NaN.
>
> status is 0 if the conversion was successful and -1 otherwise.
>
> strarray is a cell array of strings.
>
> Elements which are not defined or not valid return NaN and the
> status becomes -1.
>
> If str is a character array or a cell array of strings, then
> num and status return matrices of appropriate size.
>
> str can also contain multiple elements separated by row and
> column delimiters (cdelim and rdelim).
>
> The parameters cdelim, rdelim, and ddelim are
> optional column, row, and decimal delimiters.
>
> The default row-delimiters are newline, carriage return and semicolon
> (ASCII 10, 13 and 59). The default column-delimiters are tab, space
> and comma (ASCII 9, 32, and 44). The default decimal delimiter is
> . (ASCII 46).
>
> cdelim, rdelim, and ddelim must contain only nul,
> newline, carriage return, semicolon, colon, slash, tab, space, comma,
> or ()[]{} (ASCII 0, 9, 10, 11, 12, 13, 14, 32, 33, 34, 40,
> 41, 44, 47, 58, 59, 91, 93, 123, 124, 125).
>
> Examples:
>
> ```
>           str2double ("-.1e-5")
>           => -1.0000e-006
>           
>           str2double (".314e1, 44.44e-1, .7; -1e+1")
>           =>
>              3.1400    4.4440    0.7000
>            -10.0000       NaN       NaN
>           
>           line = "200, 300, NaN, -inf, yes, no, 999, maybe, NaN";
>           [x, status] = str2double (line)
>           => x =
>               200   300   NaN  -Inf   NaN   NaN   999   NaN   NaN
>           => status =
>                 0     0     0     0    -1    -1     0    -1     0
> ```
>
>
>
>
> **See also:** str2num.

— Function File: **strjust** (s, ["left"|"right"|"center"])  
> Shift the non-blank text of s to the left, right or center of
> the string. If s is a string array, justify each string in the
> array. Null characters are replaced by blanks. If no justification
> is specified, then all rows are right-justified. For example:
>
> ```
>           strjust (["a"; "ab"; "abc"; "abcd"])
>                => ans =
>                      a
>                     ab
>                    abc
>                   abcd
> ```

— Function File: **str2num** (s)  
> Convert the string (or character array) s to a number (or an
> array). Examples:
>
> ```
>           str2num("3.141596")
>                => 3.141596
>           
>           str2num(["1, 2, 3"; "4, 5, 6"]);
>                => ans =
>                   1  2  3
>                   4  5  6
> ```
>
> **Caution:** As `str2num` uses the `eval` function
> to do the conversion, `str2num` will execute any code contained
> in the string s. Use `str2double` instead if you want to
> avoid the use of `eval`.
>
>
>
> **See also:** str2double, eval.

— Mapping Function: **toascii** (s)  
> Return ASCII representation of s in a matrix. For example,
>
> ```
>           toascii ("ASCII")
>                => [ 65, 83, 67, 73, 73 ]
> ```
>
>
>
>
> **See also:** char.

— Mapping Function: **tolower** (s)  
— Mapping Function: **lower** (s)  
> Return a copy of the string or cell string s, with each upper-case
> character replaced by the corresponding lower-case one; non-alphabetic
> characters are left unchanged. For example,
>
> ```
>           tolower ("MiXeD cAsE 123")
>                => "mixed case 123"
> ```
>
>
>
>
> **See also:** toupper.

— Built-in Function: **toupper** (s)  
— Built-in Function: **upper** (s)  
> Return a copy of the string or cell string s, with each lower-case
> character replaced by the corresponding upper-case one; non-alphabetic
> characters are left unchanged. For example,
>
> ```
>           toupper ("MiXeD cAsE 123")
>                => "MIXED CASE 123"
> ```
>
>
>
>
> **See also:** tolower.

— Built-in Function: **do\_string\_escapes** (string)  
> Convert special characters in string to their escaped forms.

— Built-in Function: **undo\_string\_escapes** (s)  
> Converts special characters in strings back to their escaped forms. For
> example, the expression
>
> ```
>           bell = "\a";
> ```
>
> assigns the value of the alert character (control-g, ASCII code 7) to
> the string variable `bell`. If this string is printed, the
> system will ring the terminal bell (if it is possible). This is
> normally the desired outcome. However, sometimes it is useful to be
> able to print the original representation of the string, with the
> special characters replaced by their escape sequences. For example,
>
> ```
>           octave:13> undo_string_escapes (bell)
>           ans = \a
> ```
>
> replaces the unprintable alert character with its printable
> representation.
